# MicroRNA-650 Was a Prognostic Factor in Human Lung Adenocarcinoma and Confers the Docetaxel Chemoresistance of Lung Adenocarcinoma Cells via Regulating Bcl-2/Bax Expression

**DOI:** 10.1371/journal.pone.0072615

**Published:** 2013-08-21

**Authors:** Jia-Yuan Huang, Shi-Yun Cui, Yi-Tian Chen, Hai-Zhu Song, Gui-Chun Huang, Bing Feng, Ming Sun, Wei De, Rui Wang, Long-Bang Chen

**Affiliations:** 1 Department of Medical Oncology, Jinling Hospital, School of Medicine, Nanjing University, Nanjing, P. R. China; 2 Department of Biochemistry and Molecular Biology, Nanjing Medical University, Nanjing, P. R. China; Vanderbilt University Medical Center, United States of America

## Abstract

Increasing evidence shows that dysregulation of microRNAs (miRNAs) is involved in malignant transformation. We investigated the clinical significance of miR-650 and its involvement in chemoresistance to docetaxel. Our results showed that the relative expression level of miR-650 was significantly higher in LAD tissues than in corresponding nontumor tissues and high level of miR-650 expression was found to be significantly associated with high incidence of lymph node metastasis, advanced clinical stage and poor prognosis of LAD patients. Univariate and multivariate analyses indicated that high miR-650 expression was an independent prognostic factor for survival. Also, we found that the level of miR-650 in LAD tissues was correlated with the response of patients to docetaxel-based chemotherapy. Silencing of miR-650 could increase the in vitro sensitivity of docetaxel-resistant LAD cells to docetaxel, while upregulation of miR-650 decreased the sensitivity of parental LAD cells to docetaxel both *in vitro* and *in vivo*. Additionally, silencing of miR-650 could enhance the caspase-3-dependent apoptosis, which might be correlated with the decreased ratio of Bcl-2/Bax. Further researches suggested that inhibitor of growth 4 (ING4) was a direct target of miR-650. Downregulated or upregulated ING4 expression could partially rescue the effects of miR-650 inhibitor or mimics in docetaxel-resistant or parental LAD cells. Furthermore, we found that ING4 was upregulated in docetaxel-responding LAD tissues, and its expression was inversely correlated with miR-650. Thus, miR-650 is a novel prognostic marker in LAD and its expression is a potential indicator of chemosensitivity to docetaxel-based chemotherapy regimen.

## Introduction

Lung cancer is one of the commonest malignant malignancies all over the world [1]. The estimated incidence of new cases of lung cancer in the world is nearly 1,000,000 every year. In China, about 300,000 new lung cancer patients and more than 250,000 deaths from the disease are predicted each year. Adenocarcinoma of the lung (LAD) is the most common type of lung cancer and accounts for 30 to 35 percent of primary lung tumors [2]. Lung carcinogenesis is a complex, stepwise process that involves an accumulation of genetic and epigenetic changes that lead to alterations in normal lung epithelium, to in situ carcinoma, and finally to invasive and metastatic cancers [3]. Thus, elucidation of the molecular mechanisms involved in the pathogenesis of LAD will be helpful for the development of better prognostic markers and novel therapeutic targets to improve clinical treatment of LAD patients.

MicroRNAs (miRNAs) are a class of short (20-23 nucleotides in length), endogenous, single-stranded RNAs that regulate gene expression [4]. Mature miRNAs and Argonaute (Ago) proteins form the RNA-induced silencing complex (RISC), which mediates post-transcriptional gene silencing through induction of messenger RNA (mRNA) degradation or translational inhibition [5,6]. Although they account for only a minor fraction of the expressed genome, miRNAs have been found to play vital roles in various cellular processes such as growth, differentiation, apoptosis, motility and malignant transformation [7–9]. Increasing evidence shows that miRNAs are grossly dysregulated in human cancers including LAD, and may serve as oncogenes or tumour suppressors [10,11]. A single miRNA can have multiple targets involved in different oncogenic pathways, while a single gene can also be regulated by several miRNAs [12,13]. Thus, identification of dysregulated miRNAs and their target genes is critical to understand the characterization of the miRNA pathways and their underlying molecular mechanisms, which will help us to further understand the pathogenesis of LAD and to develop miRNA-direct therapeutics against this disease, including reversing tumor chemoresistance.

In this study, we determined the expression of miR-650 in LAD tissues, and found that miR-650 was upregulated in LAD tissues and correlated with advanced clinical stage and a higher incidence of lymph node metastasis in LAD patients. Also, high miR-650 expression was a poor prognostic factor for LAD patients. Furthermore, we have investigated the functional role for miR-650 in LAD cells, and showed that miR-650 could contribute to docetaxel chemoresistance of LAD cells by post-transcriptionally downregulating ING4.

## Materials and Methods

### Cell culture

Two human LAD cell lines (SPC-A1 and NCI-H1299) were purchased from the Tumor Cell Bank of Chinese Academy of Medical Science (Shanghai, China). The docetaxel-resistant SPC-A1 and NCI-H1299 cell lines (SPC-A1/DTX and H1299/DTX) were established and preserved in our lab. All the cell lines were cultured in RPMI 1640 medium containing 10% fetal bovine serum and ampicillin and streptomycin at 37°C in a humidified atmosphere of 95% air and 5% CO_2_.

### Tissue samples

A total of 96 primary LADs were collected from the Department of Cardiothoracic Surgery, Jinling Hospital between 2003 and 2006. The study was approved by the Ethic Committee of Jinling Hospital and it was performed in compliance with the Helsinki Declaration. All patients did not receive chemotherapy or radiotherapy prior to surgery. Patient characteristics are shown in Table 1. Disease histology was determined in accordance to the criteria of the World Health Organization. Pathologic staging was performed in accordance to the current International Union Against Cancer tumor-lymph node-metastasis classification. 26 randomly selected LAD tumors and their matched histologically normal lung parenchyma adjacent to the tumors (within 1 cm of the discrete tumor margin) were immediately frozen in liquid nitrogen and stored at -70°C until use. Also, 44 tissues were collected from patients with advanced LAD who received docetaxel-based chemotherapy. Patients met all of the following criteria: patients who suffered from primary LAD; a histological diagnosis of LAD with at least one measurable lesion; a clinical stage of IIIB~IV; first-line chemotherapy either with docetaxel 75 mg/m^2^ and cisplatin 100 mg/m^2^ or docetaxel 75 mg/m^2^ and carboplatin AUC 6.0 mg/ml min administered every 3 weeks for a maximum of 6 cycles; and availability of sufficient tumor tissue in paraffin blocks for assessment by immunohistochemistry. Tumor response was examined by computed tomography and evaluated according to the Response Evaluation Criteria in Solid tumors (RECIST) as complete response (CR), partial response (PR), stable disease (SD), or progressive disease (PD). All tissue samples were snap-frozen in liquid nitrogen, which were transferred to 500 µl TRIzol solution (Invitrogen, CA, USA) immediately after harvesting in order to avoid mRNA degradation. Written informed consent was obtained from all patients according to the guidelines approved by the institutional research board.

**Table 1:** Correlation between miR-650 expression and clinicopathological factors of LAD patients.

Clinicopathological factors	High miR-650 expression (n=52)	Low miR-650 Expression (n=44)	*P* value
	N (%)	N (%)	
Gender			0.439
Male	34 (65.4)	32 (72.7)	
Female	18 (34.6)	12 (27.3)	
Age			0.861
≤55	21 (40.4)	17 (38.6)	
>55	31 (59.6)	27 (61.4)	
Smoking condition			0.484
Nonsmokers	11 (21.1)	12 (28.6)	
Smokers	41 (78.9)	32 (71.4)	
Tumor differentiation			0.184
Well	9 (17.3)	3 (6.8)	
Moderate	17 (32.7)	12 (27.3)	
Poor	26 (50.0)	29 (65.9)	
T-primary tumor			0.851
T1+T2	25 (19.5)	22 (80.0)	
T3+T4	27 (80.5)	22 (20.0)	
Lymph node status			0.023^*^
N0	21 (29.3)	28 (48.8)	
N1+N2+N3	31 (70.7)	16 (51.2)	
Clinical stage			0.019^*^
I+II	23 (24.4)	30 (60.0)	
III+IV	29 (75.6)	14 (40.0)	

N, number; ^*^ statistically significant difference (*P*<0.05).

### Ethics statement

The study was approved by the Ethic Committee of Nanjing University and it was performed in compliance with the Helsinki Declaration. Written informed consent was obtained for all patient samples. All experimental animals were housed under specific pathogen-free conditions. All experimental procedures were approved by the Institutional Review Board of the Nanjing University. All procedures were performed in accordance with the Nanjing University Guide for the Care and Use of Laboratory Animals formulated by the National Society for Medical Research.

### Immunohistochemistry

Paraffin-embedded, formalin-fixed tissues were immunostained for ING4 using a rabbit anti-ING4 polyclonal antibody (Santa Cruz) at 1:500 dilution. Five-micron tissue slides from tumor tissue were de-paraffinized using xylene. Heat-mediated antigen retrieval was performed using citrate buffer (BioGenex Laboratories, San Ramon, CA). Antibody staining was visualized with DAB (Sigma, D-5637) and hematoxylin counterstain. Semi-quantitative IHC detection was used to determine the ING4 protein levels. Using the H-score method, we multiplied the percentage score by the staining intensity score. The percentage of positively stained cells was scored as ‘‘0’’ (0%), ‘‘1’’ (1%-25%), ‘‘2’’ (26%-50%), ‘‘3’’ (51%-75%) and ‘‘4’’ (76%-100%). Intensity was scored as 0: negative, 1: weak, 2: moderate, and 3: strong. The median H-score was chosen as cutoff point to separate ‘‘high ING4 expression’’ from ‘‘low ING4 expression’’ tumor samples. Immunohistochemical scoring was performed without prior knowledge of the clinical response.

### Plasmids, siRNA, miRNA mimics and miRNA inhibitors

The miR-650 expression plasmid was generated by cloning the genomic pre-miR-650 gene, with a 300-bp sequence on each flanking side, into pLMP vector (Open Biosystems Inc.) to generate plasmid pLMP-miR-650. miR-650 mimics and inhibitors (anti-miR-650) were purchased from Shanghai Gene-Pharma Co (Shanghai, China), along with the negative controls (miR-NC mimics or anti-miR-NC). DNA vector (pcDNA/ING4) expressing ING4 and the negative control vector (pcDNA/control) were previously constructed and preserved in our lab. ING4-siRNA (sc-60850) named siRNA/ING4 and non-specific control siRNA (siRNA/NC) were purchased from santa cruz biotechnology, inc (CA, USA). To construct a luciferase reporter vector, ING4 3’-UTR fragment containing putative binding sites for miR-650 was amplified by PCR using the following primers: sense 5’-CGCTCGAGAGTGGGCAGAGGAATGCCTG-3’; reverse 5’-GCGGATCCGAAGAGGTCTGGGGGACTGC-3’, and cloned downstream of the luciferase gene in the pLUC Luciferase vector (Ambion, USA) and named ING4 3’-UTR(wt). Site-directed mutagenesis of the miR-650 target-site in the ING4-3’-UTR was performed using the Quick-change mutagenesis kit (Stratagene, Heidelberg, Germany) and named ING4 3’-UTR (mut), in which ING4 3’-UTR (wt) was used as a template. For the mutated construct, the miR-650 target site tgcctcca was substituted with a acgagaa fragment.

### Transfection of oligonucleotides or plasmids and stable selection

An miR-650 mimic (100 nM) was transfected into SPC-A1 or H1299 cells, and miR-650 inhibitor (anti-miR-650, 200nM) was transfected into SPC-A1/DTX or H1299/DTX cells using Lipofectamine 2000 (Invitrogen, Carlsbad, CA, USA) according to the manufacturer’s instruction. All DNA plasmids (pLMP-miR-650 or pLMP-miR-NC) for transfection were extracted by DNA Midiprep or Midiprep kit (Qiagen). The SPC-A1 cells were transfected with those recombinant DNA vectors containing a puromycin selection marker and were selected with 5.0 µg/mL of puromycin (Sigma, USA) for 14 days. Then, single clones were isolated and expanded in media containing 2.5 µg/mL of puromycin.

### RNA extraction and real-time quantitative RT-PCR (qRT-PCR) assay

Total RNA was extracted from the tissues or cultured cells using Trizol (Invitrogen, CA, USA) according to the manufacturer’s protocol, reverse transcribed using Taqman™ microRNA reverse transcription kit and subjected to real-time PCR using TaqMan™ MicroRNA Assay kit (Applied Biosystems, USA) according to the manufacturer’s instructions. Reactions were performed using Stratagene Mx3000 instrument in triplicate. MiRNA expression was normalized to U6 snRNA.

### Western blot assay

Cell protein lysates were separated in 10% SDS polyacrylamide gels, electrophoretically transferred to polyvinylidene difluoride membranes (Roche), then detected with anti-cleaved caspase-3 or pro-caspase-3, ING4, Bcl-2 and Bax proteins. Protein loading was estimated using mouse anti-GAPDH monoclonal antibody. Lab Works^TM^ Image Acquisition and Analysis Software (UVP, Upland, CA, USA) were used to quantify band intensities. Antibodies were purchased from Univ-bio Inc (Shanghai, China).

### MTT assay

The cells were seeded into 96-well culture plates. After overnight incubation, cells treated with various concentrations of chemotherapeutic drugs. Following incubation for 24h, cell growth was measured following addition of 0.5 mg/ml MTT solution (Sigma). About 4 h later, the medium was replaced with 100µL dimethylsulfoxide (DMSO, Sigma) and vortexed for 10 min. Absorbance (A) was then recorded at 490 nm using a microplate reader (Bio-Rad, USA).

### In vitro chemosensitivity assay

The single-cell suspensions were prepared and dispersed in 96-well plates. After incubation for 72 h with the docetaxel compounds (Sigma, Saint Louis, MO, USA), the MTT (Sigma, USA) solution (0.5 mg/ml) was added. Following incubation for 4 h, 100 µL of extraction buffer were added to each well. After an overnight incubation, absorbance at 490 nm was measured using using a microplate reader (Bio-Rad, Model 680).

### Colony formation assay

The transfected LAD cells were placed in a fresh six-well plate and maintained in DMEM containing 10% FBS. 24h later, the medium was replaced with new medium. After 14 days, cells were fixed with methanol and stained with 0.1% crystal violet. Visible colonies were manually counted.

### Detection of caspase-3 activity

The activity of caspase-3 was determined using the colorimetric CaspACE Assay System (Promega Corp., Madison, WI) following the manufacturer’s instructions. Each determination was performed in triplicate.

### Flow cytometric detection of apoptosis

An annexin V-fluorescein isothiocyanate (FITC) apoptosis detection kit (Oncogene Research Products, Boston, MA) was used to detect apoptosis according to the manufacturer’s instructions. All of the samples were assayed in triplicate.

### Hoechst staining assay

The cells were cultured on 6-well tissue culture plates to confluence, and Hoechst 33342 (Sigma, USA) was added to the culture medium of living cells; changes in nuclear morphology were detected by fluorescence microscopy using a filter for Hoechst 33342 (365 nm).

### Detection of luciferase activity

Human docetaxel-resistant LAD cells (SPC-A1/DTX or H1299/DTX) grown in a 48-well plate were cotransfected with miRNA mimics/inhibitors, and pLUC firefly luciferase vectors containing empty, wild-type or mutant ING4 3’-UTR sequence using lipofectamine 2000 (Invitrogen, Carlsbad, CA). Co-transfected with two days post-transfection, the cells were collected and lysed for measurement of luciferase activities using the Dual-Luciferase Assay kit (Promega). Relative luciferase activity was calculated by normalizing the ratio of Firefly/Renilla luciferase to that of negative control-transfected cells.

### In vivo chemosensitivity assay

Animal studies were performed according to institutional guidelines. The mock or stably transfected SPC-A1 cells (SPC-A1/pLMP-miR-650 and SPC-A1//pLMP-miR-NC) were suspended in 100 µL PBS and injected subcutaneously into the right side of the posterior flank of female BALB/c athymic nude mice (Department of comparative medicine, Jinling Hospital, Nanjing, China) at 5 to 6 weeks of age. When the average tumor size reached about 50 mm^3^, docetaxel was given through intraperitoneal injection with a concentration of 1.0 mg/kg, one dose every other day with 3 doses totally. Tumor growth was examined weekly for at least 5 wk. After 35 days, the mice were killed, necropsies were performed, and tumors were weighted. Tumor volumes were calculated by using the equation V (in mm^3^) = *A*×*B* ^2^/2, where *A* is the largest diameter, and *B* is the perpendicular diameter.

### Bioinformatic and statistical analysis

The online miRNA databases (TargetScan, miRBase, and PicTarget) were used for prediction of miR-650 target genes. The SPSS10.0 program was used for general statistical and survival analysis. Experimental data were expressed as the mean±SEM. For comparison of means between two groups, a two-tailed *t*-test was used, and for comparison of means among three groups, one-way ANOVA was used The Spearman correlation test was used for analyses of primary tumors. Survival probabilities were determined using Kaplan-Meier analysis and the significance of difference was analyzed by a log-rank test. Significance was accepted at *P*<0.05.

## Results

### MiR-650 was significantly upregulated in LAD tissues

**Figure 1:**
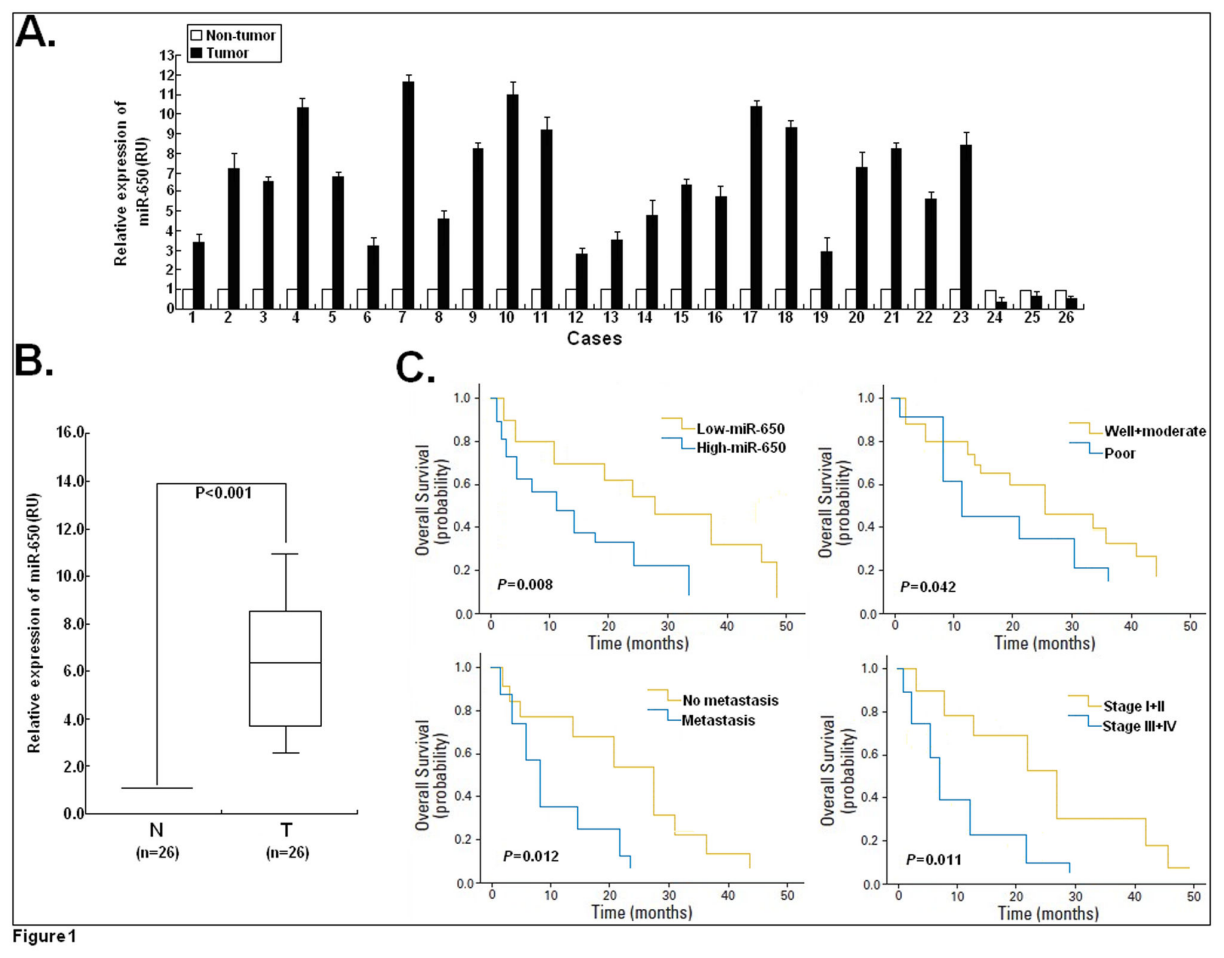
Detection of miR-650 expression in LAD tissues and corresponding nontumor tissues. (**A**) Upregulation and downregulation of miR-650 expression. qRT-PCR for miR-650 was performed by using 26 human LAD tissues and corresponding adjacent normal tissues. The mean and standard deviation of expression levels relative to U6 expression levels are shown and are normalized to the expression in the normal tissue of each matched pair. RU, relative units. (**B**) The mean level of miR-650 expression in LAD tissues (n=26) was significantly higher than that in corresponding adjacent normal tissues (n=26) (*P*<0.001). The data represent at least triplicate measurements from single RNA samples. (**C**) Kaplan-Meier survival analysis for LAD patients. Comparison of the overall survival (OS) between low-miR-650(n=44) and high-miR-650(n=52) patients (*P*=0.008). Comparison of the OS between patients with tumor well and moderate differentiation (n=41) and patients with tumor poor differentiation (n=55; *P*= 0.042). Comparison of the OS between patients with tumor metastasis (n=47) and patients with no tumor metastasis (n=49; *P*=0.012). Comparison of the OS between clinical stage I+II (n=53) and III+IV patients (n=43; *P*=0.011). *P*<0.05 indicates a significant difference between groups. Corresponding *P* values analyzed by log-rank tests are indicated.

To gain insight into the biological role of miR-650 in human LAD progression, we first detected the expression of miR-650 in 26 human LAD tissues and corresponding non-tumor tissues by qRT-PCR (Figure 1A). Compared with corresponding non-tumor tissues, 23 of 26 human LAD tissues (88.5%) showed that miR-650 was significantly upregulated 11.6-fold to 2.5-fold, but only 3 cases (11.5%) were downregulated in different degree. In general, the mean expression level of miR-650 in LAD tissues was significantly higher than that in corresponding non-tumor tissues (*P*<0.01; Figure 1B). Thus, these data indicated that upregulation of miR-650 might be, at least in part, involved in human LAD development.

### Association of miR-650 expression with clinicopathological factors of LAD patients

To further analyze the association of miR-650 expression with clinicopathological factors of LAD patients, qRT-PCR assay was performed to detect miR-650 expression in a total of 96 LAD cases. Then, according to the median miR-650 expression level (median=8.45, normalized to U6 snRNA), LAD cases were classified into two expression groups: high-miR-650 expression group (n=52) and low-miR-650 expression group (n=44). By statistical analysis (Table 1), it was shown that the level of miR-650 expression was significantly correlated with lymph node metastasis and clinical stage of LAD patients (*P*=0.023 and 0.019, respectively), suggesting that high miR-650 expression group showed a higher incidence of lymph node metastasis (70.7%) and more advanced clinical stage (75.6%) than the low miR-60 expression group (51.2% and 40.0%, respectively). However, there were no significant differences between miR-650 expression and other clinicopathological facors, including gender, age, smoking condition, tumor differentiation and T-primary tumor (*P*=0.439, 0.861, 0.484, 0.502 and 0.851, respectively).

### MiR-650 expression was an independent prognostic factor for LAD patients

To explore the association of miR-650 expression with the clinical outcome of LAD patients, the overall survival of LAD patients relating to miR-650 expression, status of tumor differentiation, lymph node metastasis and clinical stage was determined (Figure 1C). LAD patients with high-miR-650 expression (n=52) showed a significantly worse prognosis than those patients with low-miR-650 expression (n=44). The median survival time for the LAD patients with high-miR-650 was 12.5 months (95% CI: 9.5-15.7 months), and for LAD patients with low-miR-650 was 28.3 months (95% CI: 17.5-36.2 months; *P*=0.008). Additionally, other factors (tumor differentiation, lymph node metastasis and clinical stage) were shown to be closely correlated with the survival of LAD patients. LAD patients with well or moderate differentiation (n=41) had a longer survival time than those patients with poor differentiation (n=55; median: 26.5 months vs 10.3 months, *P*=0.042). Also, LAD patients with lymph node metastasis (n=47) had a significantly shorter survival time than those without lymph node metastasis (n=49; median: 9.7 months vs 31.2 months, *P*=0.012). Moreover, LAD patients with clinical stage III/IV (n=43) had a significantly shorter survival time than those with clinical stage I/II (n=53; median: 9.7 months vs 31.2 months, *P*=0.012).

To further investigate whether miR-650 expression might be a prognostic factor in LAD patients, univariate and multivariate data analyses were performed using the Cox proportional hazards regression model (Table 2). By univariate analysis, lymph node metastasis (N_1_+N_2_+N_3_/N_0_), clinical stage (III+IV/I+II) and miR-650 expression (High/Low) were the prognostic factors to predict a poor prognosis (*P*=0.026, 0.012 and 0.008, respectively). By multivariate analysis of the prognostic factors, we confirmed that high miR-650 expression was an independent prognostic factor (unfavorable; risk ratio: 4.65; 95% confidence interval: 1.58-6.21; *P*=0.011), while lymph node metastasis was also an independent prognostic factor (*P*=0.028). These data strongly suggested that the miR-650 expression in LAD patients was closely related to a poor prognosis.

**Table 2:** Cox’s regression model analysis of prognostic factors in LAD patients.

		Univariate analysis			Multivariate analysis	
Variables	Unfavorable / Favorable	HR (95% CI)	*P*-value		HR (95% CI)	*P*-value
Gender	Male/female	0.78 (0.61-1.44)	0.608			
Age	≥55/<55	1.22 (0.83-3.15)	0.433			
Smoking condition	Smokers/Nonsmokers	1.06 (0.57-2.69)	0.187			
Tumor differentiation	Poor/Well+Moderate	0.95 (0.46-1.84)	0.215		1.53 (0.88-3.15)	0.372
T-primary tumor	T3+T4/T1+T2	2.12 (0.91-3.47)	0.088		2.51 (0.74-3.53)	0.187
Lymph node metastasis	N_1_+N_2_+N_3_/N_0_	3.27 (1.64-5.11)	0.026^*^		2.13 (0.74-3.14)	0.214
Clinical stage	III+IV/I+II	4.45 (2.79-7.33)	0.012^*^		1.73 (1.23-2.94)	0.028^*^
MiR-650 expression	High/Low	3.46 (1.48-4.38)	0.008^*^		4.65 (1.58-6.21)	0.011^*^

* *P*<0.01. Abbreviations: HR, hazard ratio; 95% CI, 95% confidence interval.

### Expression of miR-650 was negatively correlated with responses of LAD patients to docetaxel

**Figure 2:**
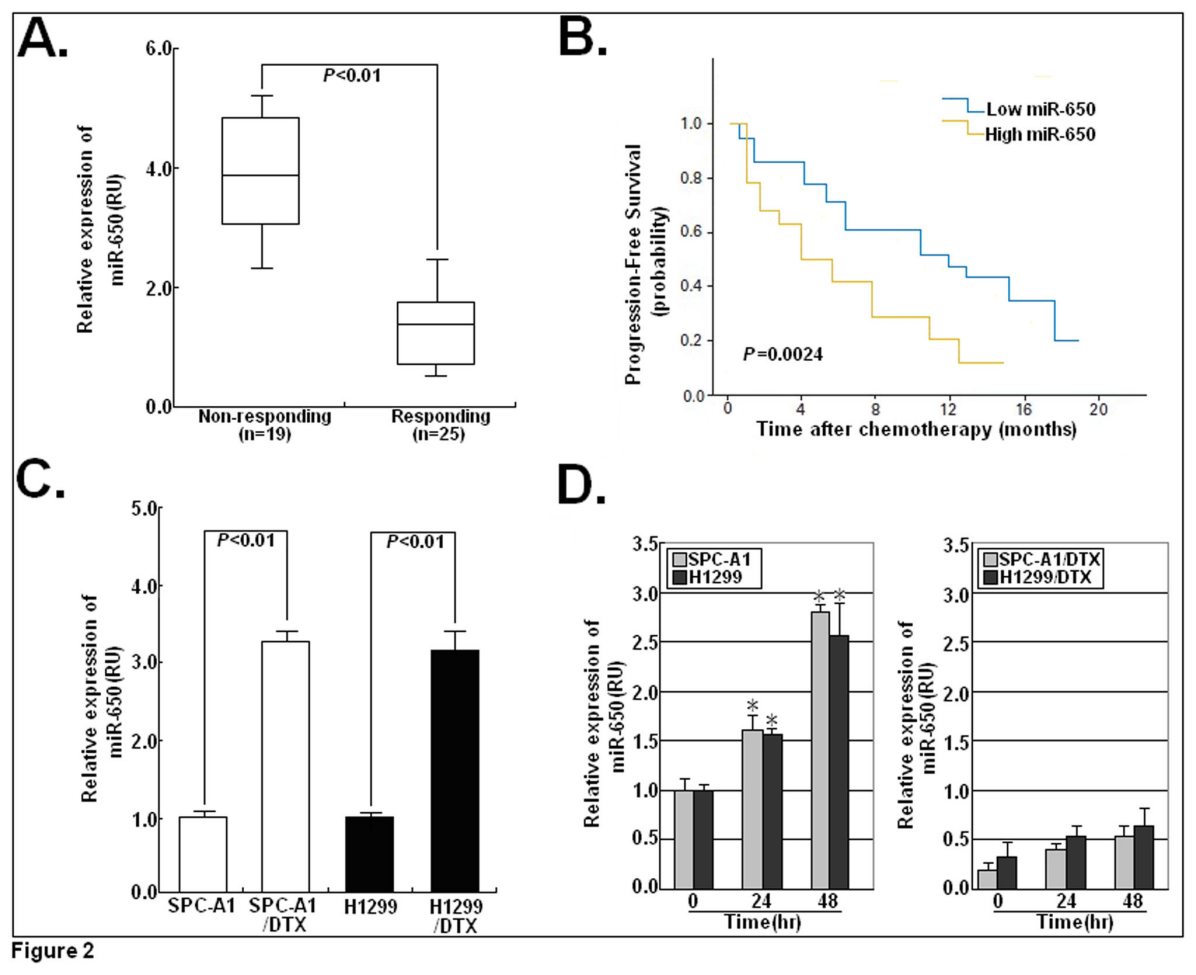
Correlation between miR-650 expression and the responses of LAD patients to docetaxel. (**A**) Relative expression levels of miR-650 was detected in docetaxel-sensitive (n=25) and insensitive (n=19) LAD tissues via qRT-PCR. (**B**) Kaplan-Meier survival curve indicates that patients with high miR-650 expression have shorter progress-free survival (PFS) than those with low miR-650 expression (log-rank test, *P*=0.0024). (**C**) Detection of miR-650 expression in docetaxel-resistant LAD cells (SPC-A1/DTX and H1299/DTX) and parental LAD cells (SPC-A1 and H1299) via qRT-PCR. (**D**) Analysis of the expression of miR-650 in docetaxel-resistant or parental LAD cells after treatment with docetaxel (5.0 µg/L) via qRT-PCR. Abundance of miRNA was normalized to U6 RNA. Each experiment was performed at least in triplicate.

To explore the correlation between miR-650 expression and the efficiency of chemotherapy, we analyzed the survival data in patients who received docetaxel-based adjuvant chemotherapy (n=44). Tumors were divided into two groups: responding (CR+PR) and non-responding (SD+PD). Our data showed that the mean level of miR-650 expression in non-responding tumor tissues (n=19) was significantly higher than that in responding tumor tissues (n=25) (*P*<0.001; Figure 2A). Then, ROC curve analysis was performed to establish the optimal cutoff value for the HSCORE of miR-650 expression level, which yielded a value of 143.5 (data not shown). Patients with a low level of miR-650 expression (HSCORE < 143.5) had a significantly longer progression-free survival (PFS) than did those with a high level of miR-650 expression (HSCORE ≥ 143.5) (*P*=0.0024; Figure 2B). Thus, it was concluded that the expression of miR-650 in advanced LAD tissues might be negatively correlated with the response of patients to docetaxel-based chemotherapy.

### Downregulation of miR-650 reverses the resistance of docetaxel-resistant LAD cells to docetaxel

Previously, two docetaxel-resistant LAD cell lines (SPC-A1/DTX and H1299/DTX) were developed from the docetaxel-nonresistant LAD cell lines (SPC-A1 and H1299) in our lab, respectively [14]. To further investigate whether miR-650 is an important regulator of acquired docetaxel resistance in LAD cells, qRT-PCR was performed to detect the expression of miR-650 in docetaxel-resistant and parental LAD cells. As shown in Figure 2C, the level of miR-650 expression in SPC-A1/DTX or H1299/DTX cells was significantly higher than that in parental SPC-A1 or H1299 cells (*P*<0.05). Next, we testify whether docetaxel treatment could affect the expression of miR-650 in LAD cells. The miR-650 levels after treatment with docetaxel (5.0 µg/L) were increased in the parental SPC-A1 or H1299 cells, but not in the SPC-A1/DTX or H1299/DTX cells (Figure 2D).

**Figure 3:**
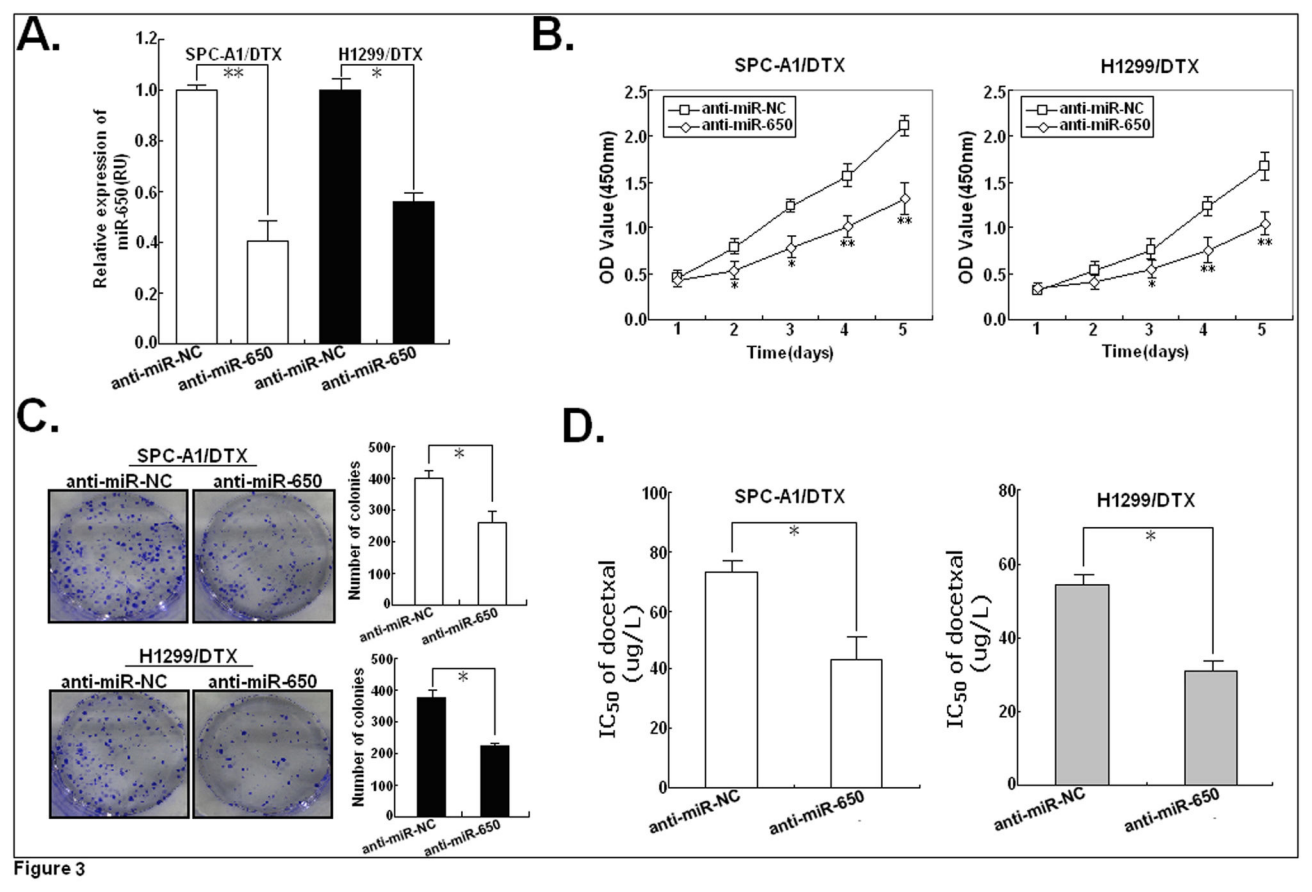
Effect of anti-miR-650 on the in vitro sensitivity of docetaxel-resistant LAD cells to docetaxel. (**A**) 48h after SPC-A1/DTX or H1299/DTX cells were transfected with anti-miR-650 (or anti-miR-NC), qRT-PCR assay was performed to detect the expression of miR-650. (**B**) MTT analysis of growth in anti-miR-650 or anti-miR-NC-transfected SPC-A1/DTX or H1299/DTX cells. (**C**) Representative results of colony formation of SPC-A1/DTX or H1299/DTX cells transfected with anti-miR-650 or anti-miR-NC. (**D**) Analysis of the IC_50_ values of docetaxel in SPC-A1/DTX or H1299/DTX cells transfected with anti-miR-NC or anti-miR-650 using MTT assays. Results represent the average of three independent experiments (mean±SD). *P<0.05 or **P<0.01, in comparison with anti-miR-NC-transfected cells.

In order to examine the correlation between the upregulation of miR-650 and the resistance of LAD cells to docetaxel, miR-650 inhibitor was transfected into docetaxel-resistant LAD cells. 48h after transfection, qRT-PCR assay showed that the expression of miR-650 in anti-miR-650-transfected SPC-A1/DTX or H1299/DTX cells was significantly decreased compared with that in anti-miR-NC-transected cells (*P*<0.05; Figure 3A). MTT assay was performed to detect the effect of miR-650 expression on the growth of docetaxel-resistant LAD cells. Compared with anti-miR-NC, anti-miR-650 could induce the decreased growth of SPC-A1/DTX or H1299/DTX cells in a time-dependent manner (Figure 3B). Meanwhile, anti-miR-650 could lead to the decreased colony formation capacity of SPC-A1/DTX or H1299/DTX cells (*P*<0.05; Figure 3C). Next, we analyzed the changes of docetaxel sensitivity of chemoresistant LAD cells induced by anti-miR-650. Compared with anti-miR-NC-transfected cells, the IC_50_-value of docetaxel in anti-miR-650-transfected SPC-A1/DTX or H1299/DTX cells was significantly decreased by approximately 40.2% or 43.2%, respectively (*P*<0.05; Figure 3D). Thus, downregulation of miR-650 could reverse the resistance of docetaxel-resistant LAD cells to docetaxel by inhibiting cell growth.

### Upregulation of miR-650 reduces the in vitro sensitivity of parental LAD cells to docetaxel

**Figure 4:**
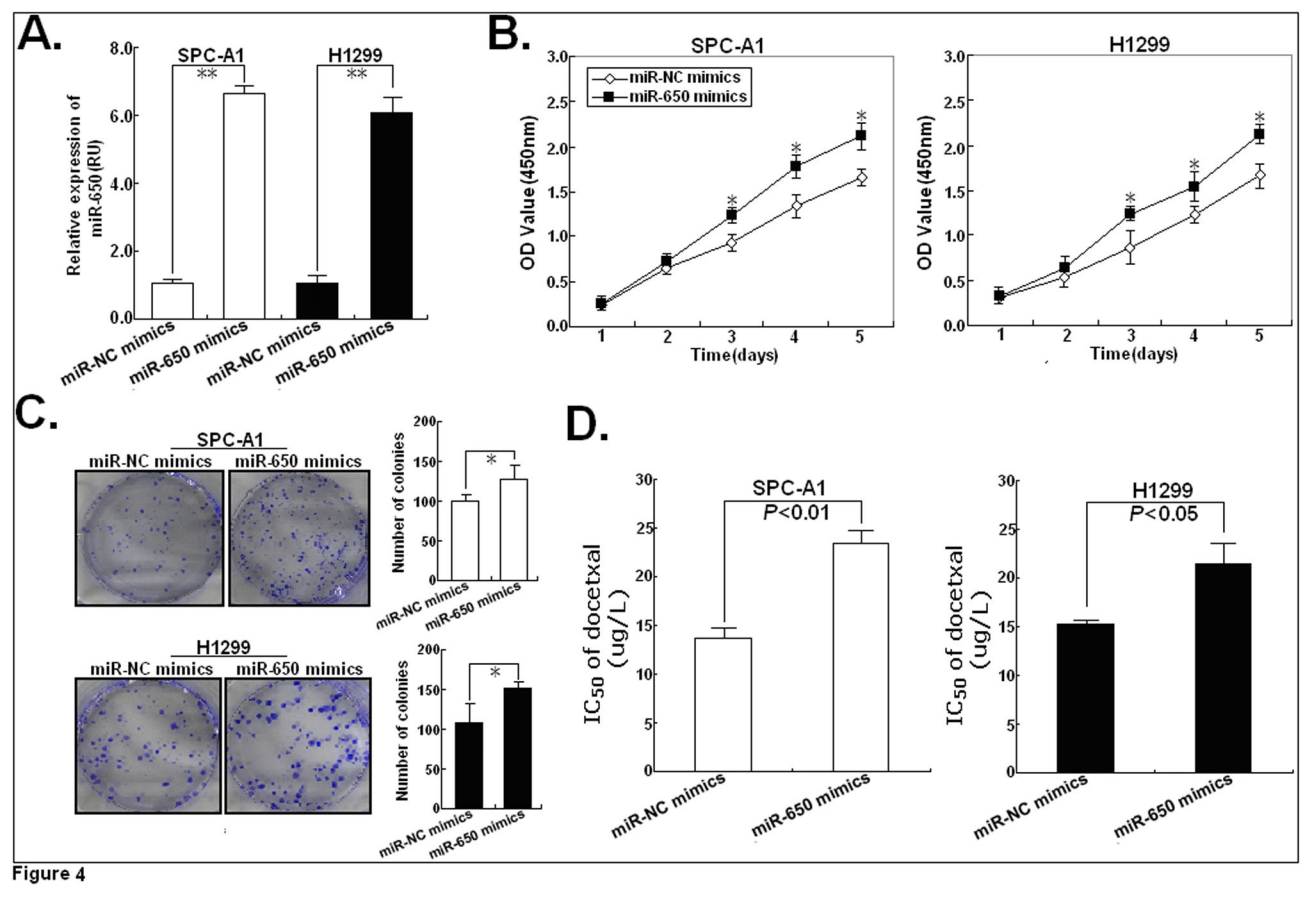
Effect of miR-650 mimics on the in vitro sensitivity of parental LAD cells to docetaxel. (**A**) 48h after SPC-A1 or H1299 cells were transfected with miR-650 mimics or miR-NC mimics, qRT-PCR assay was performed to detect the expression of miR-650. (**B**) MTT analysis of growth in SPC-A1 or H1299 cells transfected with miR-650 mimics or miR-NC mimics. (**C**) Representative results of colony formation of SPC-A1 or H1299 cells transfected with miR-650 mimics or miR-NC mimics. (**D**) Analysis of the IC_50_ values of docetaxel in SPC-A1 or H1299 cells transfected with miR-650 mimics or miR-NC mimics using MTT assay. Results represent the average of three independent experiments (mean±SD). *P<0.05 or **P<0.01, in comparison with anti-miR-NC-transfected cells.

Next, we investigated whether upregulation of miR-650 could affect the sensitivity of LAD cells to docetaxel. Then, miR-650 mimics was transfected into parental SPC-A1 or H1299 cells. 48h after transfection, qRT-PCR assay showed that the expression of miR-650 in miR-650 mimics-transfected SPC-A1/DTX or H1299/DTX cells could be significantly increased (*P*<0.01; Figure 4A). Results of MTT assay indicated that miR-650 mimics could induce the increased growth ability of parental SPC-A1 or H1299 cells compared with miR-NC mimics (Figure 4B). Likewise, the ability of colony formation in SPC-A1 or H1299 cells transfected with miR-650 mimics was increased compared with cells transfected with miR-NC mimics (*P*<0.05; Figure 4C). More importantly, compared with miR-NC mimics-transfected cellsh, the IC_50_-value of docetaxel in miR-650 mimics-transfected SPC-A1 or H1299 cells could be increased by approximately 72.3% or 41.1%, respectively (Figure 4D). These data suggested that upregulation of miR-650 might be an important inducer of docetaxel resistance of LAD cells.

### ING4 was a functional target of miR-650 in docetaxel-resistant LAD cells

**Figure 5:**
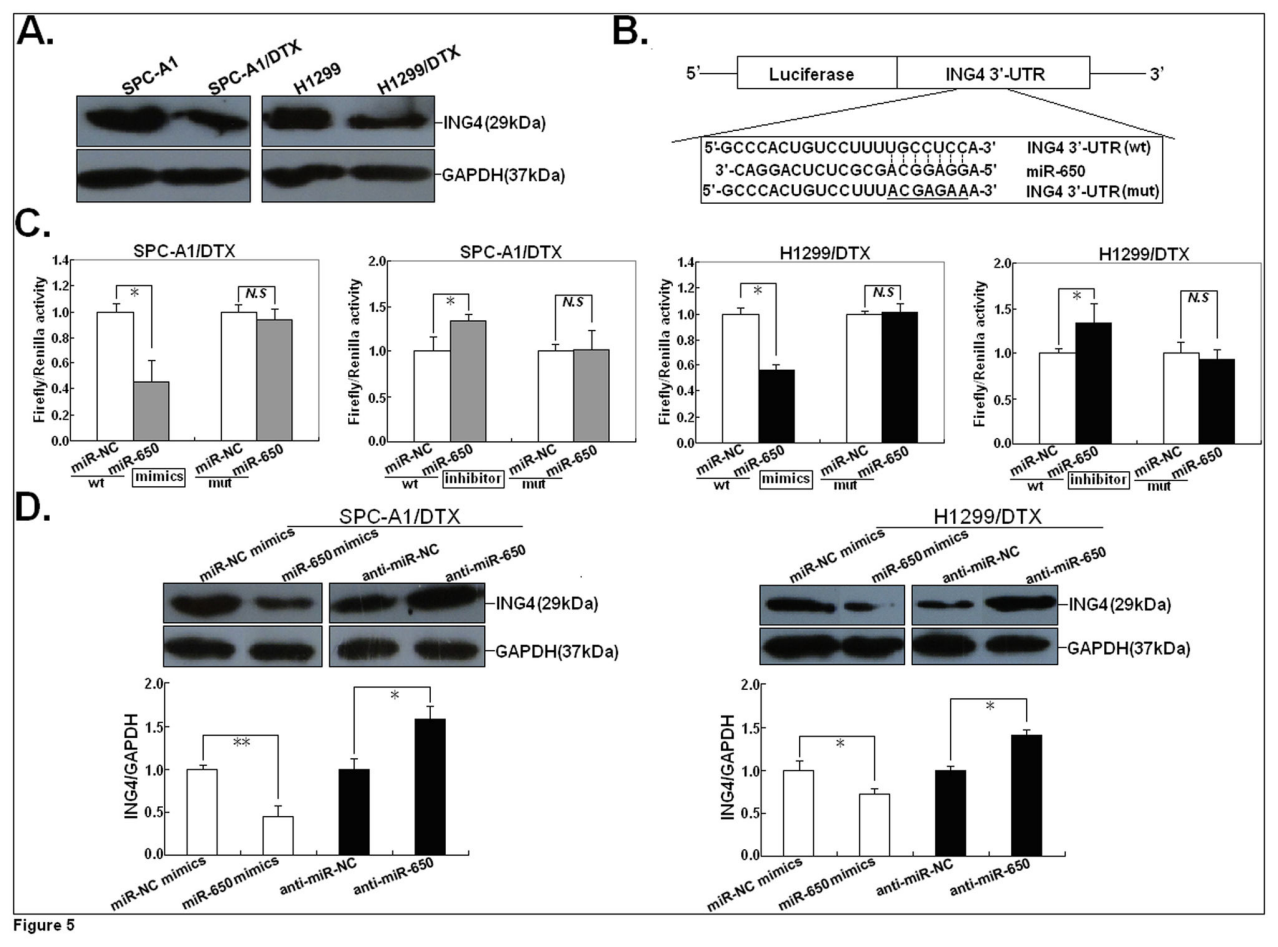
ING4 is a direct target of miR-650. (**A**) Western blot analysis of ING4 protein expression in docetaxel-resistant and parental LAD cells. (**B**) Sequence of miR-650 binding site in the ING4 3’ UTR predicted with TargetScan, miRBase and PicTarget and the 3’-UTR region of ING4 mRNA is partially complementary to miR-650. (**C**) SPC-A1/DTX or H1299/DTX cells were co-transfected with miR-650 mimics or inhibitor and pLUC vector with ING4 3’-UTR-wt or mut. After 24 hours, the luciferase activity was measured. Values are presented as relative luciferase activity after normalization to Renilla luciferase activity. (**D**) Western Blot analysis of ING4 protein expression in SPC-A1/DTX or H1299/DTX cells transfected with miR-650 mimics (or miR-NC mimics) or anti-miR-650 (or anti-miR-NC). The data are expressed as the mean value ± SEM of the results obtained from three independent experiments. *P<0.05 or **P<0.01, in comparison with anti-miR-NC or miR-NC mimics-transfected cells.

Previously, we have reported that downregulation of inhibitor of growth 4 (ING4) was involved in docetaxel resistance of LAD cells [14]. However, the molecular mechanisms involved in downregulation of ING4 remain unclear. As miRNAs function mainly through the inhibition of target genes, the targets of miR-650 that function in docetaxel-resistant LAD cells were further investigated. The targets of miR-650 were predicted through at least three databases (Pictarget, miRBas and TargetScan), and ING4 was selected as a putative target, which was also recently reported in gastric cancer [16]. The 3’-untranslated region (UTR) of ING4 gene stood out because of the presence of one evolutionarily conserved binding site (260~266bp) for miR-650, suggesting cooperative binding and biologically effective interactions. First, we detected the expression of ING4 protein in parental and docetaxel-resistant LAD cells, and showed that the expression of ING4 protein in SPC-A1/DTX or H1299/DTX cells was significantly downregulated compared with that in parental SPC-A1 or H1299 cells (Figure 5A). Then, to further confirm target specificity between miR-650 and ING4 in LAD cells, we performed luciferase reporter assay with a vector containing the putative ING4 3’-UTR target site downstream of the luciferase reporter gene, which was transfected into LAD cells. Base pairing between miR-650 and wild-type (wt) or mutant (mut) target site in the 3’-UTR of ING4 mRNA was shown in Figure 5B. Luciferase activities of LAD cells transfected with ING4-wt construct were significantly lower after transfection of miR-650 mimics and were significantly higher after transfection of anti-miR-650, whereas those with ING4-mut construct showed no significant difference (Figure 5C). Next, we analyzed the effect of miR-650 on ING4 protein expression, and found that miR-650 mimics could lead to the decreased expression of ING4 protein in SPC-A1/DTX or H1299/DTX cells, while anti-miR-650 could induce the increased expression of ING4 protein (Figure 5D).

**Figure 6:**
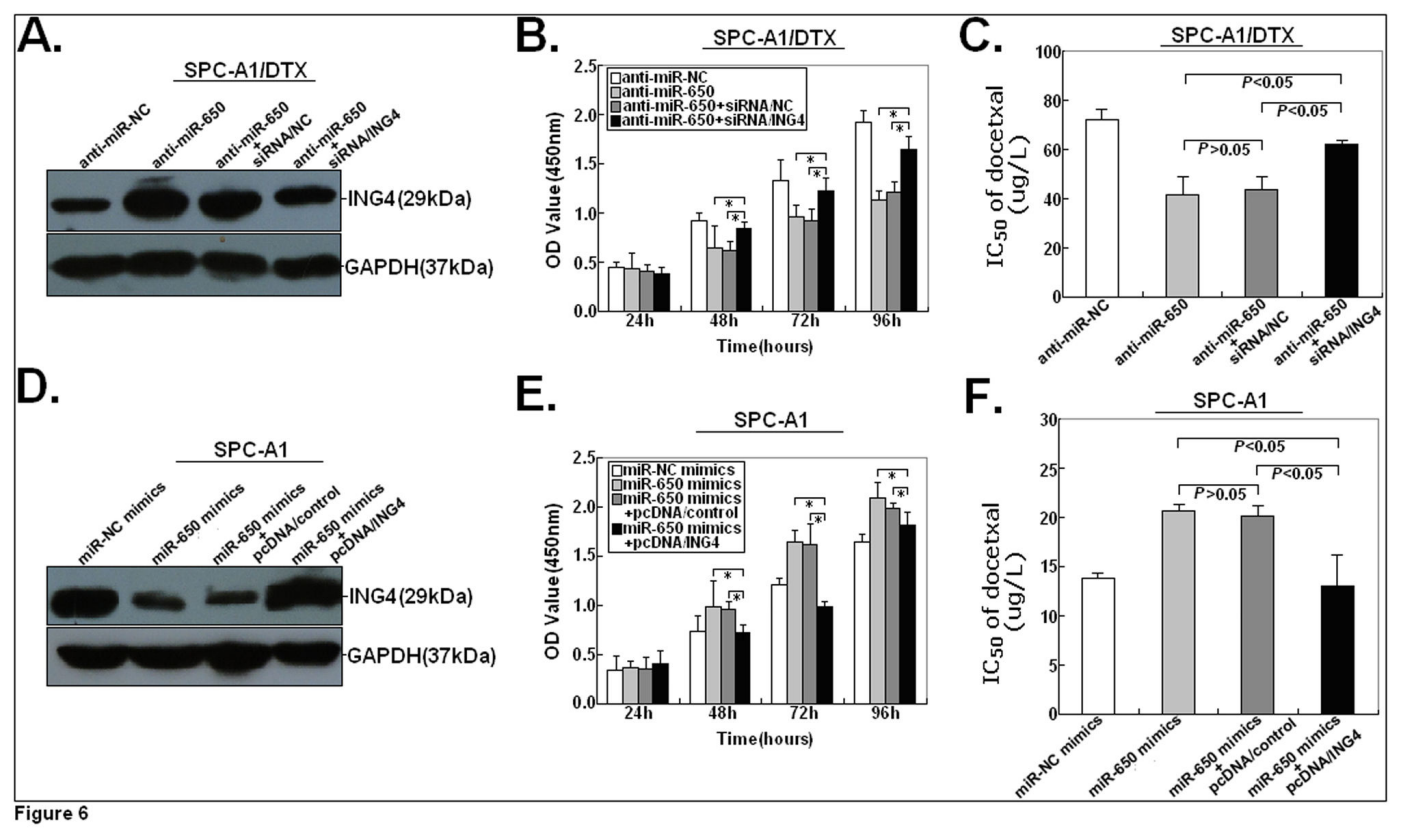
siRNA/ING4 or pcDNA/ING4 could partially rescue the effects of anti-miR-650 or miR-650 mimics on the sensitivity of SPC-A1/DTX or SPC-A1 cells to docetaxel. (**A**) Western blot analysis of ING4 protein expression in anti-miR-NC or anti-miR650-transfected SPC-A1/DTX cells or SPC-A1/DTX cells co-transfected with anti-miR650 and siRNA/NC or siRNA/ING4. (**B**) MTT analysis of growth in anti-miR-NC or anti-miR650-transfected SPC-A1/DTX cells or SPC-A1/DTX cells co-transfected with anti-miR650 and siRNA/NC or siRNA/ING4. *P<0.05, in comparison with anti-miR-650-transfected cells or cells co-transfected with anti-miR650 and siRNA/NC. (**C**) Analysis of the IC_50_ values of docetaxel in anti-miR-NC or anti-miR650-transfected SPC-A1/DTX cells or SPC-A1/DTX cells co-transfected with anti-miR650 and siRNA/NC or siRNA/ING4. (**D**) Western Blot analysis of ING4 protein expression in miR-NC or miR-650 mimics-transfected SPC-A1 cells or SPC-A1 cells co-transfected with miR-650 mimics and pcDNA/control or pcDNA/ING4. (**E**) MTT analysis of growth in miR-NC or miR-650 mimics-transfected SPC-A1 cells or SPC-A1 cells co-transfected with miR-650 mimics and pcDNA/control or pcDNA/ING4. *P<0.05, in comparison with miR-650 mimics-transfected cells or cells co-transfected with miR-650 mimics and pcDNA/control. (**F**) Analysis of the IC_50_ values of docetaxel in miR-NC or miR-650 mimics-transfected SPC-A1 cells or SPC-A1 cells co-transfected with miR-650 mimics and pcDNA/control or pcDNA/ING4 using MTT assay. The data are expressed as the mean value ± SEM of the results obtained from three independent experiments.

In our previous study, we have reported that upregulation of ING4 could reverse the docetaxel resistance of docetaxel-resistant LAD cells, suggesting that upregulation of ING4 could mimic the effects of anti-miR-650 on the chemosensitivity of docetaxel-resistant LAD cells to docetaxel [15]. To further testify the roles of ING4 in miR-650-induced chemoresistance of LAD cells, siRNA/ING4 was transfected into anti-miR-650-transfected SPC-A1/DTX cells. It was found that siRNA/ING4 could reverse the increased ING4 protein expression in SPC-A1/DTX cells induced by anti-miR-650 (Figure 6A). Also, siRNA/ING4 could partially rescue the growth inhibition of SPC-A1/DTX cells induced by anti-miR-650 (Figure 6B). Meanwhile, siRNA/ING4 could also partially rescue the decreased IC_50_-value of docetaxel in SPC-A1/DTX cells induced by anti-miR-650 (Figure 6C). Next, pcDNA/ING4 vector was transfected into miR-650 mimics-transfected SPC-A1 cells. Results indicated that pcDNA/ING4 could reverse the decreased ING4 protein expression in SPC-A1 cells induced by miR-650 mimics (Figure 6D). Likewise, pcDNA/ING4 could partially rescue the growth promotion and increased IC_50_-value of docetaxel in SPC-A1 cells induced by miR-650 mimics (Figure 6E-F). Collectively, these findings further suggest that ING4 is a functional and direct target gene for miR-650 in LAD cells.

### MiR-650 and ING4 affects the apoptosis of docetaxel-resistant LAD cells by way of regulating Bcl-2/Bax expression

**Figure 7:**
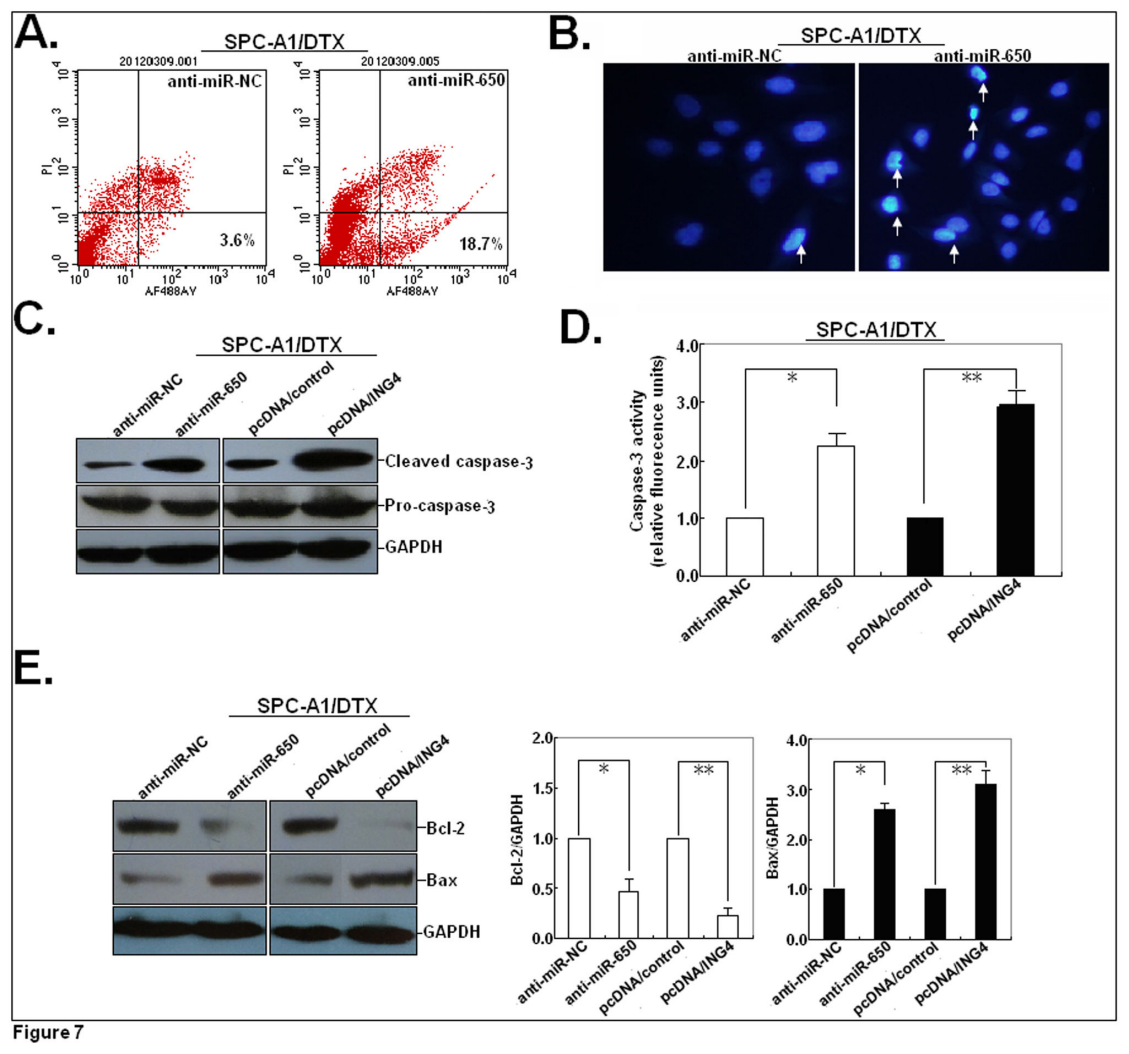
Effects of miR-650 expression on the survival pathway in SPC-A1/DTX cells. (**A**) Flow cytometric analysis of apoptosis in anti-miR-NC or anti-miR-650-transfected SPC-A1/DTX cells. (**B**) Hoechst staining analysis of apoptosis in anti-miR-NC or anti-miR-650-transfected SPC-A1/DTX cells. (**C**) Western blot detection of the expression of pro-caspase-3 and cleaved caspase-3 proteins in anti-miR-650 or pcDNA/ING4-transfected SPC-A1/DTX cells (anti-miR-NC or pcDNA/control was used as control). (**D**) Detection of caspase-3 activity in anti-miR-650 or pcDNA/ING4-transfected SPC-A1/DTX cells (anti-miR-NC or pcDNA/control was used as control). (**E**) Western blot analysis of the expression of Bcl-2 and Bax proteins in anti-miR-650 or pcDNA/ING4-transfected SPC-A1/DTX cells (anti-miR-NC or pcDNA/control was used as control). Equal loading was confirmed by showing equal GAPDH levels. Results represent the average of three independent experiments (mean±SD). *P<0.05 or **P<0.01, in comparison with anti-miR-NC or pcDNA/control-transfected cells.

As miR-650 could reverse the resistance of docetaxel-resistant LAD cells to docetaxel and we confirmed that ING4 was a direct and functional target, we investigated the molecular mechanisms by which miR-650 and ING4 mediated the regulation of chemosensitivity of LAD cells. First, we analyzed the effect of miR-650 expression on apoptosis of SPC-A1/DTX cells, and showed that anti-miR-650 could induce the increased apoptosis of SPC-A1/DTX (Figure 7A). Also, using the Hoechst staining assay, condensed and fragmented nuclei in anti-miR-650-transfected SPC-A1/DTX cells were obviously observed (Figure 7B). In previous study, we have showed that upregulation of ING4 could significantly increase the apoptosis of docetaxel-resistant LAD cells, suggesting that downregulation of miR-650 could lead to the same apoptosis-inducing effect as upregulation of ING4. Also, we found that both anti-miR-650 and pcDNA/ING4 could increase the expression of cleaved caspase-3 in SPC-A1/DTX cells but had no effects on the expression of pro-caspase-3 protein (Figure 7C). Meanwhile, compared with anti-miR-NC or pcDNA/control-transfected cells, the activity of caspase-3 in anti-miR-650 or pcDNA/ING4-transfected SPC-A1/DTX cells was increased by 152.3% or 185.8%, respectively (Figure 7D). Further, Western blot assay confirmed the decreased expression of Bcl-2 protein and the increased expression of Bax protein in anti-miR-650 or pcDNA/ING4-transfected SPC-A1/DTX cells (Figure 7E), which led to a decrease in the cell antiapoptotic ability (Bcl-2/Bax ratio).

To further investigate whether ING4 was involved in the effect of miR-650 on apoptosis in SPC-A1/DTX cells, we then performed rescue experiments. After transfected with anti-miR-650, SPC-A1/DTX cells were co-transfected with siRNA/ING4. Flow cytometric analysis showed that the co-tramsfection could partially rescue the increased apoptosis in SPC-A1/DTX cells induced by anti-miR-650 (Figure S1A). Also, the co-tramsfection could partially rescue the increased expression of cleaved caspase-3 protein and the enhanced activity of caspase-3 in SPC-A1/DTX cells induced by anti-miR-650 (Figure S1B-C). Furthermore, the co-tramsfection could partially rescue the decreased expression of Bcl-2 protein and the increased expression of Bax protein in SPC-A1/DTX cells induced by anti-miR-650 (Figure S1D). These data indicated that upregulation of ING4 was potentially involved in the apoptosis-promoting function of anti-miR-650, which could induce the caspase-3-dependent apoptosis by way of regulating the expression of Bcl-2/Bax.

### Upregulation of miR-650 reduces the in vivo sensitivity of parental LAD cells to docetaxel

**Figure 8:**
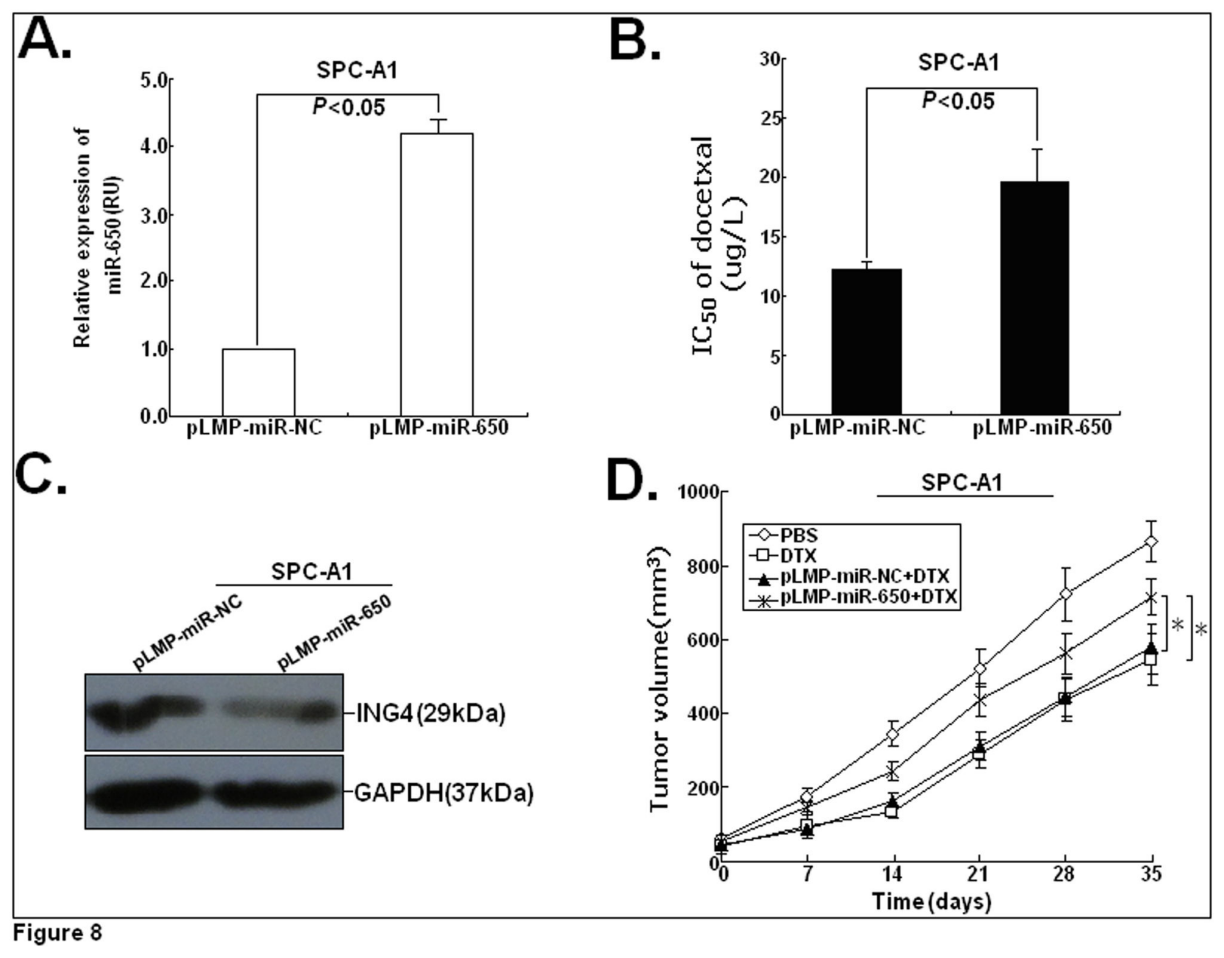
MiR-650 increases docetaxel sensitivity *in vivo*. (**A**) qRT-PCR analysis of miR-650 expression in SPC-A1/DTX cells stably transfected with pLMP-miR-NC or pLMP-miR-650. (**B**) Analysis of the docetaxel IC_50_ values in stably transfected SPC-A1/DTX cells. (**C**) Western blot analysis of ING4 protein expression in stably transfected SPC-A1/DTX cells. (**D**) SPC-A1/DTX cells stably expressing miR-650 were injected into mice subcutaneously. After the tumor was established (tumor size=50 mm^3^), mice were i.p. injected with a concentration of 1.0 mg/kg (one dose every other day with 3 doses totally) followed by monitoring of tumor size for 5 weeks (mean±SEM, n=8). *P< 0.05.

To further investigate the possible effect of miR-650 expression on LAD cell sensitivity to docetaxel *in vivo*, SPC-A1 cells were stably transfected with pLMP-miR-650 or control pLMP-miR-NC vector. As shown in Figure 8A, the expression of miR-650 was significantly upregulated in SPC-A1/pLMP-miR-650 cells compared with SPC-A1/pLMP-miR-NC cells (*P*<0.01). Upregulation of miR-650 could significantly reduce the expression of ING4 protein in SPC-A1 cells (*P*<0.05; Figure 8B). Also, the IC_50_-value of docetaxel in SPC-A1/pLMP-miR-650 cells was modestly increased by approximately 53.4% (Figure 8C). Next, s.c. tumors were induced in nude mice followed by treatment with docetaxel. Following docetaxel treatments, the tumors formed from SPC-A1/pLMP-miR-650 cells grew faster than those formed from SPC-A1/pLMP-miR-NC cells (*P*<0.05; Figure 8D). These data support the notion that miR-650 could induced the increased docetaxel resistance of LAD cells *in vivo*.

### ING4 is often downregulated in docetaxel-resistant LAD tissues and negatively correlated with miR-650 expression

**Figure 9:**
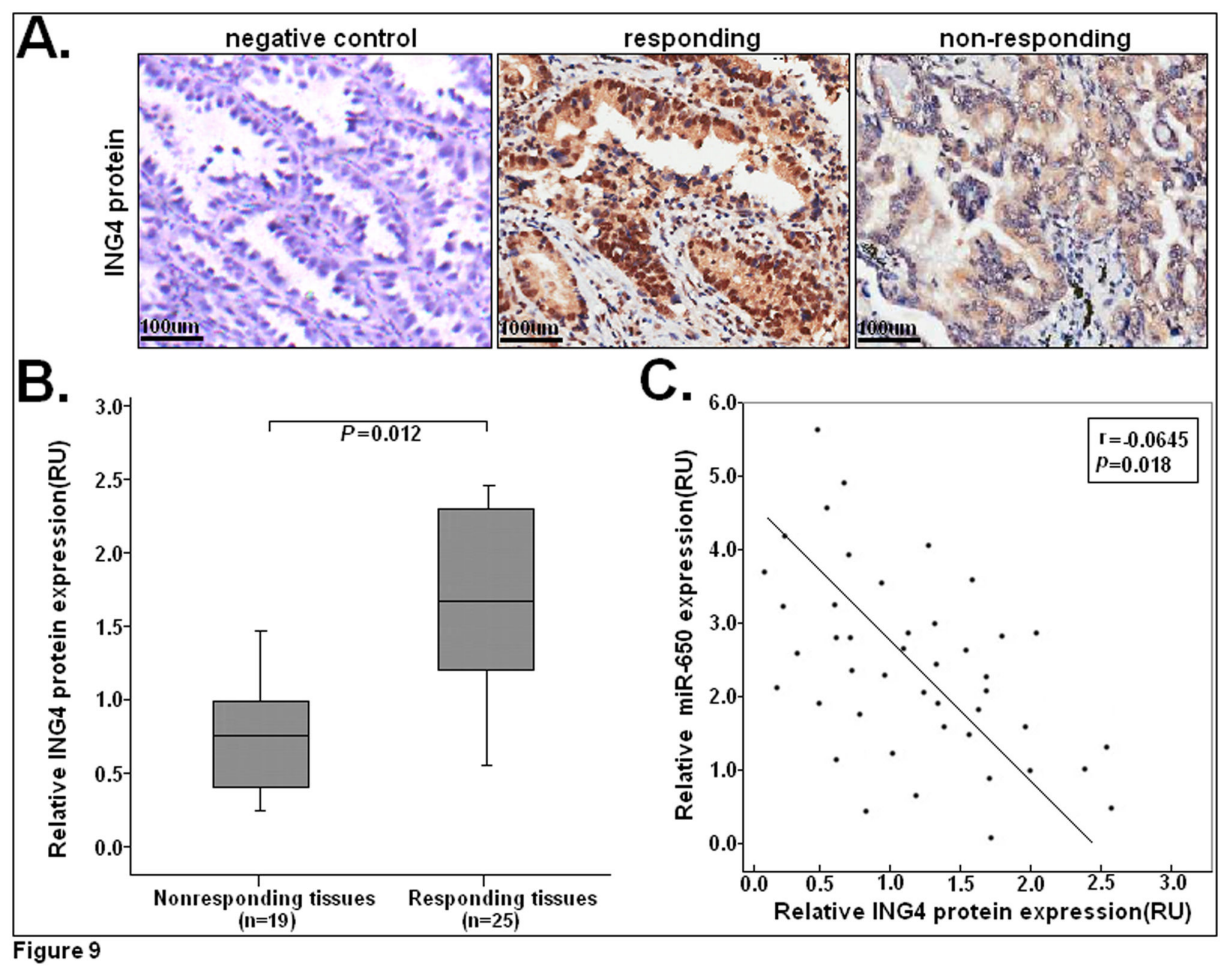
ING4 was significantly downregulated in docetaxel-non-responding LAD tissues and inversely correlated with miR-650 expression. (**A**) Immunohistochemical staining for ING4 protein in non-responding or responding LAD tissues. Isotype IgG at the same concentration was used as a negative control for immunohistochemical staining. Positive ING4 protein staining was mainly located in the nucleus of tumor cells. IN4 expression was higher in responding tumors than in nonresponding tumors. *Bars*: 100µm. (**B**) Relative expression levels of ING4 protein was detected in docetaxel-responding (n=25) and non-responding (n=19) LAD tissues via Western blot assay. GAPDH was used as an internal control. (**C**) A statistically significant inverse correlation between miR-650 and ING4 protein levels in 44 cases of LAD tissues (Spearman’s correlation analysis, r = -0.0645; *P*=0.018). Results represent the average of three independent experiments (mean±SD). Corresponding *P* values analyzed by Spearman correlation test are indicated.

Because ING4 is the target of miR-650, immunohistochemistry was performed to detect the expression of ING4 in LAD tissues collected from patients who received docetaxel-based chemotherapy (n=44). As shown in Figure 9A, ING4 expression was higher in the responding than in the non-responding LAD tissues. To further investigate the correlation of ING4 expression with the responses of LAD patients to docetaxel, Western blot assays were performed to detect the expression of ING4 protein in those tissues. In contrast to miR-650, the relative expression level of ING4 protein in responding tumor tissues was significantly higher than that in non-responding tumor tissues (*P*=0.022; Figure 9B). Further, the inverse correlation between miR-650 and ING4 protein expression was verified by linear regression analysis (Spearman rank test r=-0.645; *P*=0.018) (Figure 9C).

## Discussion

Increasing evidence suggests that in addition to protein-encoding genes, miRNAs have more than a cursory role in the pathogenesis of human cancer development [16,17]. Thus, miRNA expression signatures seem to hold great promise in tumor characterization and could be used as potential diagnostic and prognostic markers for tumor diagnosis and therapy [18]. The key finding of this study is that upregulation of miR-650 expression is correlated with LAD development and progression. Statistical analyses indicate that LAD patients with high expression of miR-650 have a poorer prognosis. Also, our results reveal that upregulation of miR-650 could induce docetaxel resistance of LAD cells via regulating Bcl-2/Bax expression by targeting tumor suppressor ING4. These data suggest that miR-650 will be a significant predictor of poor prognosis for LAD patients and be a potential target for chemosensitizing LADs.

Several recent studies have demonstrated that many miRNAs are dysregulated in human LADs. Jiang, et al conducted miRNA expression profiling in matched lung adenocarcinoma and uninvolved lung using 56 pairs of fresh-frozen and 47 pairs of formalin-fixed, paraffin-embedded samples from never smokers and showed that increased miR-708 expression in NSCLC and its association with poor survival in LAD from never smokers [19]. Rothschild and colleagues showed that both miR-381 and miR-29b repressed ID1 and were dysregulated in LAD. Kaduthanam, et al identified microRNAs (miRNAs) in serum of patients associated with early relapse in pulmonary adenocarcinoma by using qRT-PCR based low-density arrays (664 miRNAs) and showed that serum miR-142-3p was associated with early relapse in operable LAD patients [20]. Also, Patnaik and colleagues identified microRNA expression profiles of whole blood in LAD, and showed that microRNAs miR-190b, miR-630, miR-942, and miR-1284 were the most frequent constituents of the classifiers [21]. In our previous study, we have reported that miR-451 could function as a tumor suppressor not only in squamous cell carcinoma but also in lung adenocarcinoma [22]. These findings imply that miRNAs are involved in LAD development and progression and could serve as potential diagnostic markers and therapeutic targets. In the present study, we found that the average level of miR-650 expression was significantly higher in LAD tissues as compared with paired adjacent nontumor tissues. Results from quantitative analysis of qRT-PCR indicated that high level of miR-650 expression was closely correlated with advanced clinical stage and a high incidence of lymph node metastasis in LAD patients. Also, LAD patients with high-level miR-650 expression had a shorter median overall survival (12.5 months) than those with low-level miR-650 expression (28.3 months). These data, together with further univariate and multivariate analyses, suggest that high expression of miR-650 might be a significant predictor of poor prognosis for LAD patients. More importantly, further analyses showed that the expression of miR-650 was correlated with the efficiency of chemotherapy. It was found that the mean level of miR-650 expression in non-responding tumor tissues was significantly higher than that in responding tumor tissues. Additionally, the PFS of LAD patients with high-level miR-650 who received docetaxel-based chemotherapy was significantly lower than that of patients with low-level miR-650. Thus, it was concluded that overexpression of miR-650 might be involved in docetaxel-resistant phenotypes of human LADs.

Recently, the abberant expression of miR-650 has been reported to be correlated with the aggressiveness of colorectal cancer, gastric cancer, and chronic lymphocytic leukemia cells [23,24]. Feng and colleagues reported that downregulation of N-myc downstream-regulated gene 2 (NDRG2) expression involving promoter methylation and microRNA-650 promoted colorectal cancer progression [23]. In gastric cancer, Zhang’ et al reported that microRNA-650 could target ING4 to promote gastric cancer tumorigenicity [25]. Also, Mraz and colleagues showed that microRNA-650 expression is influenced by immunoglobulin gene rearrangement and affects the biology of chronic lymphocytic leukemia (CLL) [24]. In their studies, higher expression of miR-650 was found to be associated with a favorable CLL prognosis and influenced the proliferation capacity of B cells. However, there were no reports about the association of miR-650 expression with the chemoresistance of tumor cells. Previously, the multidrug-resistant LAD cell lines (SPC-A1/DTX and H1299/DTX) were established from human LAD cell lines (SPC-A1 and H1299) in our lab by stepwise selection using docetaxel as inducing reagent. Furthermore, a subset of miRNAs was found to be differentially expressed in SPC-A1/DTX cells and its parental SPC-A1 cells [26]. Our further research data showed that miR-200b or miR-100 could modulate the resistance of LAD cells to docetaxel by targeting E2F3 or Plk1 [27,28]. In the present study, we firstly showed that miR-650 was significantly upregulated in docetaxel-resistant LAD cells (SPC-A1/DTX and H1299/DTX) compared with in parental LAD cells (SPC-A1 and H1299). By modulating the miR-650 level in LAD cells, we revealed that miR-650 could mediate the docetaxel resistance of LAD cells both *in vitro* and *in vivo*. Further, we conducted luciferase reporter assay and Western blotting to confirm that ING4 was a direct and functional target of miR-650 in LAD cells. ING4, a novel member of ING family comprising six members characterized by a highly conserved C-terminal plant homedomain (PHD)-like zinc-finger domain, has been reported to be involved in a variety of human biological and pathological processes including oncogenesis, angiogenesis, and DNA repair [29,30]. The downregulation of ING4 has been found in many human cancers including gastric cancer, ovarian carcinoma, head and neck squamous cell carcinoma and lung cancer [31–34]. In our previous study, a cDNA microarray analysis was performed to compare differential gene expression profile between docetaxel-resistant and parental LAD cell lines, and further functional data suggest that ING4 could modulate the sensitivity of LAD cells to taxanes by regulating apoptosis-related proteins. It was observed that the effects of ING4 overexpression on the chemoresistance of docetaxl-resistant LAD cells were similar to the effects of miR-650 inhibitor. Furthermore, silencing of ING4 could partially rescue the effects of miR-650 inhibitor on the chemosensitivity of docetaxel-resistant LAD cells. We then further elucidated the possible mechanisms of chemosensitivity enhancement in LAD cells induced by miR-650 inhibitor or ING4 overexpression. Results from flow cytometry and Hoechst staining assays indicated that miR-650 inhibitor could induce the increased capase-3-dependent apoptosis. Previous researches have shown that ING4 could function as tumor suppressor in human cancers by regulating the Bcl-2 family proteins. In this study, it was found that docetaxel-resistant LAD cells transfected with miR-650 inhibitor presented with decreased expression of Bcl-2 protein and increased expression of Bax protein, and the decreased Bcl-2/Bax ratio led to the execution of apoptotic progression in docetaxel-resistant LAD cells. Likewise, silencing of ING4 could partially rescue the increased caspase-3-dependent apoptosis and decreased Bcl-2/Bax ratio induced by miR-650 inhibitor. At the same time, the expression of ING4 protein was negatively correlated with miR-650 expression in responding or non-responding LAD tissues. Our findings demonstrated that miR-650 confers the docetaxel chemoresistance of lung adenocarcinoma cells via regulating Bcl-2/Bax expression by targeting ING4. Of course, many studies have indicated that one miRNA could regulate multiple target genes, whereas one gene could also be regulated by multiple miRNAs. Therefore, miR-650 might regulate other target genes which are involved in formation of chemoresistant types in LAD cells, which needs to be further elucidated in future.

This study showed that high level of miR-650 expression was correlated with enhanced malignant potential and poor prognosis of LAD patients. Furthermore, miR-650 could affect the chemosensitivity of LAD cells to docetaxel via regulating Bcl-2/Bax expression by directly targeting ING4. Since the size of tissue sample in the present study is small, further investigation of a larger patient population will be necessary to confirm the association of miR-650 and its target ING4 with the responses of LAD patients to docetaxel-based chemotherapy.

## Supporting Information

Figure S1 **ING4 was involved in the effect of miR-650 on apoptosis in SPC-A1/DTX cells.** (**A**) Flow cytometry detection of apoptosis in SPC-A1/DTX cells transfected with anti-miR-650 (or anti-miR-NC) or co-transfected with anti-miR-650 and siRNA/ING4 (or siRNA/NC). (**B**) Western blot detection of the expression of pro-caspase-3 and cleaved caspase-3 proteins in SPC-A1/DTX cells transfected with anti-miR-650 (or anti-miR-NC) or co-transfected with anti-miR-650 and siRNA/ING4 (or siRNA/NC). (**C**) Detection of caspase-3 activity in SPC-A1/DTX cells transfected with anti-miR-650 (or anti-miR-NC) or co-transfected with anti-miR-650 and siRNA/ING4 (or siRNA/NC). (**D**) Western blot analysis of the expression of Bcl-2 and Bax proteins in SPC-A1/DTX cells transfected with anti-miR-650 (or anti-miR-NC) or co-transfected with anti-miR-650 and siRNA/ING4 (or siRNA/NC). Equal loading was confirmed by showing equal GAPDH levels. Results represent the average of three independent experiments (mean±SD). *P<0.05 or **P<0.01. N.S, not significant.
(TIF)
