# Supplementary figures and images for: MicroRNA-650 Was a Prognostic Factor in Human Lung Adenocarcinoma and Confers the Docetaxel Chemoresistance of Lung Adenocarcinoma Cells via Regulating Bcl-2/Bax Expression
Source: PLoS One. 2013 Aug 21;8(8):e72615. doi: 10.1371/journal.pone.0072615 (PMC3749147; doi:10.1371/journal.pone.0072615)

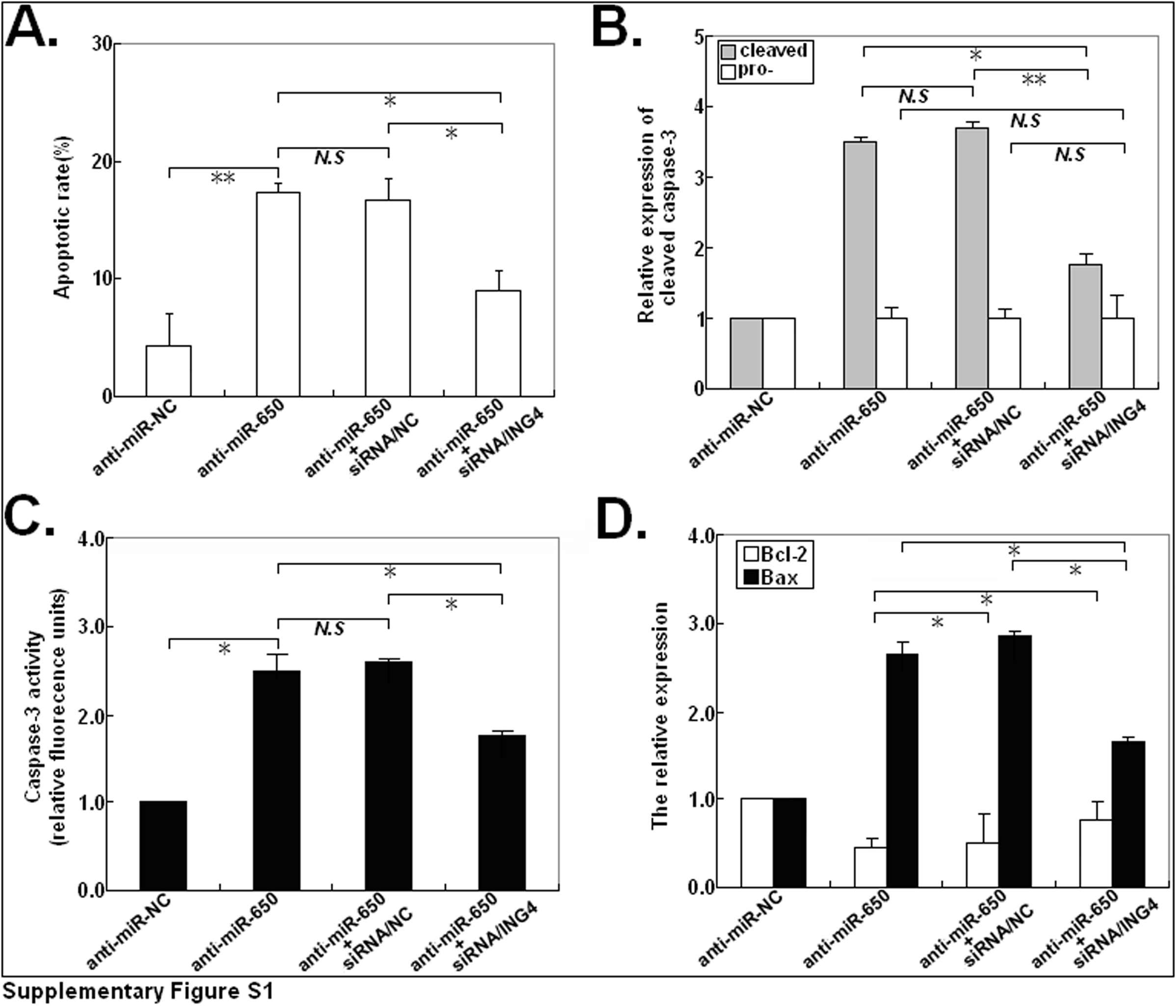

Supplement: Figure S1 — ING4 was involved in the effect of miR-650 on apoptosis in SPC-A1/DTX cells. (A) Flow cytometry detection of apoptosis in SPC-A1/DTX cells transfected with anti-miR-650 (or anti-miR-NC) or co-transfected with anti-miR-650 and siRNA/ING4 (or siRNA/NC). (B) Western blot detection of the expression of pro-caspase-3 and cleaved caspase-3 proteins in SPC-A1/DTX cells transfected with anti-miR-650 (or anti-miR-NC) or co-transfected with anti-miR-650 and siRNA/ING4 (or siRNA/NC). (C) Detection of caspase-3 activity in SPC-A1/DTX cells transfected with anti-miR-650 (or anti-miR-NC) or co-transfected with anti-miR-650 and siRNA/ING4 (or siRNA/NC). (D) Western blot analysis of the expression of Bcl-2 and Bax proteins in SPC-A1/DTX cells transfected with anti-miR-650 (or anti-miR-NC) or co-transfected with anti-miR-650 and siRNA/ING4 (or siRNA/NC). Equal loading was confirmed by showing equal GAPDH levels. Results represent the average of three independent experiments (mean±SD). *P<0.05 or **P<0.01. N.S, not significant. (TIF) [file pone.0072615.s001.tif]
